# Regulation of PKM2 expression and function by GLIS3 during metabolic reprogramming in polycystic kidneys

**DOI:** 10.1038/s12276-026-01676-5

**Published:** 2026-03-13

**Authors:** Justin B. Collier, Hong Soon Kang, Sara A. Grimm, Tanushree Mukherjee, Chitrangda Srivastava, Anton M. Jetten

**Affiliations:** 1 Cell Biology Group, Immunity, Inflammation and Disease Laboratory, National Institute of Environmental Health Sciences, National Institutes of Health, Research Triangle Park, NC USA; 2 Integrative Bioinformatics, National Institute of Environmental Health Sciences, National Institutes of Health Research, Research Triangle Park, NC USA

**Keywords:** Transcriptomics, Polycystic kidney disease, Mechanisms of disease, Gene regulation

## Abstract

Deficiency in GLI-similar 3 (GLIS3) in humans and mice causes polycystic kidney disease (PKD). We previously reported that *Glis3-*knockout (KO) kidneys undergo metabolic reprogramming and remain more reliant on aerobic glycolysis. Here, to understand the mechanism by which GLIS3 controls this process, we analyzed its role in regulating glycolytic gene expression using transcriptomics and cistromics. Transcriptomic analysis revealed increased expression of key glycolytic genes and reduced expression of several gluconeogenic genes in *Glis3*-KO compared with wild-type kidneys. Chromatin immunoprecipitation followed by sequencing analysis showed that many of these genes were directly regulated by GLIS3 in coordination with the transcription factor, HNF-1B. *Pkm* emerged as a key upregulated glycolytic GLIS3 target gene, which via alternative splicing generates two protein isoforms, PKM1 and PKM2. Expression of *Pkm* and the dimeric form of PKM2, which promotes aerobic glycolysis, was elevated in *Glis3*-KO kidneys, whereas exogenous GLIS3 expression in primary *Glis3*-KO renal epithelial cells suppressed *Pkm* expression. Phosphorylation of PKM2 at Y105 and S37, which stimulate dimer formation, nuclear localization and aerobic glycolysis, is increased in *Glis3*-KO kidneys, consistent with a role for PKM2 in metabolic reprogramming and cyst formation. Consistent with this, siRNA-mediated knockdown of PKM2 in *Glis3*-KO renal epithelial cells suppressed spheroid growth and glycolytic activity. In addition, pharmacological inhibition of PKM2 with compound 3K in GLIS3-deficient spheroid cultures and *Glis3*-KO kidneys significantly reduced spheroid size, cyst number and cystic area. Our study identifies GLIS3 as a novel regulator of glycolysis and PKM2 function, whose dysregulation contributes to metabolic reprogramming and cystogenesis in *Glis3*-KO kidneys.

## GLIS3 deficiency drives metabolic changes in kidney disease

The study explores the role of a protein called GLIS3 in kidney function. When GLIS3 does not work properly, it can lead to a serious kidney condition called autosomal recessive polycystic kidney disease, which causes cysts in the kidneys and ultimately renal failure. Researchers found that GLIS3 is important for controlling how cells in the kidney use energy. Researchers discovered that these mice had more kidney cysts and relied more on a process called aerobic glycolysis. They used various methods, including genetic analysis and experiments with kidney cells, to study this process. They found that the protein PKM2 was more active in these mice, which might be causing the cysts to grow. By reducing PKM2 activity using a specific inhibitor, they were able to decrease the size and number of cysts in the mice.

This summary was initially drafted using artificial intelligence, then revised and fact-checked by the author.

## Introduction

The Krüppel-like zinc finger transcription factor, GLI-similar 3 (GLIS3), plays a critical role in the regulation of biological processes in various tissues, including pancreas, thyroid, testis and kidney^1–4^. Loss of GLIS3 function in humans and mice leads to a multiorgan phenotype that includes neonatal diabetes, congenital hypothyroidism and autosomal recessive polycystic kidney disease (ARPKD)^1,2,5–9^. ARPKD is a rare and severe form of cystic kidney disease that is manifested prenatally and during early childhood^10^. In GLIS3-deficient mice, renal cysts are observed perinatally and steadily increase in size and number postnatally leading to loss of normal kidney functions and end-stage renal disease^3,8,11,12^. These observations underscore the critical role of GLIS3 in regulating normal kidney physiology.

The first month of normal postnatal kidney development is accompanied by notable alterations in gene expression and functions. This includes changes in energy metabolism and an increased reliance on oxidative phosphorylation. In polycystic kidney disease (PKD), this shift in energy metabolism is dysregulated and renal cells remain more dependent on aerobic glycolysis^13–22^. Recently, we demonstrated that GLIS3 deficiency in mice disrupts this critical metabolic transition by suppressing the increase in mitochondrial-related gene expression, including those involved in oxidative phosphorylation and the tricarboxylic acid cycle^11^. Seahorse and metabolic analyses indicated decreased use of mitochondrial respiration along with increased glucose use and lactate production, supporting the conclusion that kidneys from GLIS3-deficient mice remain more reliant on aerobic glycolysis^11^.

These observations suggested that GLIS3 may also function as a critical regulator of glycolysis and glycolytic gene transcription. Hexokinase (HK), phosphofructokinase 1 (PFK1) and pyruvate kinase (PKM) are critical in regulating glycolysis. PKM, which catalyzes the conversion of phosphoenolpyruvate into pyruvate, is the rate-limiting enzyme during the last stage of glycolysis and has been shown to have critical roles in the reprogramming of cell metabolism during kidney development, PKD and tumorigenesis^23,24^. The *Pkm* gene undergoes alternative splicing to produce two transcripts encoding the isoforms PKM1 and PKM2^24–26^. PKM2 enzymatic activity is regulated by whether it exists as a monomer, dimer or tetramer and by specific posttranslational modifications^23,24,27^. The tetrameric form stimulates the generation of adenosine triphosphate via oxidative phosphorylation, whereas PKM2 phosphorylated on either serine 37 (pS37) or tyrosine 105 (pY105) promotes aerobic glycolysis and lactate production and has been associated with metabolic reprogramming and increased proliferation^28–32^.

Here, we investigated the role of GLIS3 in the regulation of glycolysis, glycolytic gene expression and PKM2 activity in kidneys of GLIS3-deficient mice, with emphasis on their relationship to metabolic reprogramming in ARPKD. We demonstrate that several glycolytic genes, including *Pkm*, are directly regulated by GLIS3 in coordination with the hepatocyte nuclear factor 1β (HNF-1B). We further show that the levels of PKM2(pS37) and PKM2(pS107) are elevated in GLIS3-deficient kidneys compared with wild-type (WT) kidneys, resulting in higher PKM2 dimer formation. This increase in phosphorylated PKM2 and its dimeric form probably promotes aerobic glycolysis, enhanced cell proliferation and cystogenesis in GLIS3-deficient kidneys. This is supported by data showing that siRNA-mediated knockdown of PKM2 in GLIS3-deficient renal epithelial cells (RECs) reduced spheroid growth, lactate production and glycolytic activity. Similarly, the PKM2 inhibitor, compound 3K, significantly inhibited cyst formation in spheroids derived from GLIS3-deficient renal cells and decreased both the size and number of renal cysts in GLIS3-deficient kidneys. In summary, our study identifies GLIS3 as a new transcriptional regulator of glycolytic gene expression, including *Pkm2*. Our findings highlight the important function of PKM2 in the regulation of energy biosynthesis and in the progression of cystogenesis in ARPKD, underscoring its potential as a therapeutic target.

## Material and methods

### Mice

Ubiquitous GLIS3-deficient *Glis3*-KO2 mice (C57BL/6-*Glis3*<tm3(mCherry)Amj>) and *Glis3*-EGFP mice (C57BL/6-*Glis3*<tm3(*Glis3*-EGFP)Amj>) expressing a GLIS3–EGFP fusion protein were described previously^6^. Tissue-selective *Glis3*-Pax8Cre mice (B6;129-*Glis3*<tm2Amj> Pax8<tm1.1(cre)Mbu>) were generated by crossing *Glis3*fl/fl mice with Pax8Cre mice (B6.129P2(Cg)-Pax8tm1.1(cre)Mbu/J; Jackson Laboratory)^33^. Mice were backcrossed onto C57BL/6 for at least seven generations. Mice were routinely fed an NIH-31 diet (ND; Harlan) and killed using CO_2_. In all mouse experiments, WT littermates/age-matched mice were used as controls. To examine the effect of PKM2 inhibition on cyst formation, PND7 *Glis3*-Pax8Cre mice were treated intraperitoneally with the PKM2 inhibitor, compound 3K (S8616; Selleckchem Chemicals) (10 mg/kg) or vehicle control (10% dimethylsulfoxide (DMSO), 90% corn oil) every 24 h for 7 days. All animal studies followed guidelines outlined by the National Institutes of Health (NIH) Guide for the Care and Use of Laboratory Animals, and protocols were approved by the Institutional Animal Care and Use Committee at the National Institute of Environmental Health Sciences (NIEHS).

### RNA-seq and ChIP-seq data analysis

Transcriptome analysis was performed using our RNA sequencing (RNA-seq) data from kidneys of PND28 *Glis3*-KO2 mice and littermate controls that were deposited under accession no. GSE240074^11^. For cistrome analysis, GLIS3 and HNF-1B chromatin immunoprecipitation followed by sequencing (ChIP-seq) data, deposited under accession nos. GSE240074 and GSE240072, were used^11^. In brief, kidneys from *Glis3*-EGFP and WT mice were homogenized and homogenate crosslinked in 1% formaldehyde for 10 min and the reaction subsequently quenched by the addition of 125 mM glycine for 10 min. The crosslinked cells were washed two times with PBS, resuspended in lysis buffer A for 10 min, pelleted and resuspended in lysis buffer B for 10 min. Samples were subsequently sheared in lysis buffer C for 40 min using an S220 focused-ultrasonicator (Covaris). After centrifugation, the cleared chromatin supernatant was incubated with an anti-GFP (ab290; Abcam) or anti-HNF-1B (720259; ThermoFisher) antibody for ChIP. After subsequent washes, ChIPed-DNA was eluted and amplified. Libraries were synthesized using a NEXTflex Rapid DNA-Seq kit (PerkinElmer). Sequencing was performed with NextSeq 500 or MiSeq (Illumina). The sashimi plots demonstrating splice junctions were generated with splicejam R package in R4.3.2 as previously described^34^.

### RT–qPCR

Total RNA was isolated from renal tissue or cultured renal primary cells using the PureLink RNA Mini Kit (ThermoFisher) according to the manufacturer’s protocol, and cDNA was generated using a High-Capacity cDNA Reverse Transcription kit (Applied Biosystems). Real-time quantitative PCR with reverse transcription (RT–qPCR) analysis was performed in triplicate using SYBR Green with StepOnePlus Real Time PCR System (Applied Biosystems) as described previously^11,35^. All results were normalized to *Actb* expression, and the relative fold change in the expression of each gene was determined by the 2^ΔΔCt^ method. Primer sequences are presented in Supplementary Table 1.

### Cystic index and number

Cross-sections of frozen OCT-embedded whole kidneys from vehicle-treated and compound 3K-treated *Glis3*-KO2 mice were imaged using an EVOS M7000 imager (Invitrogen), and quantification of cyst number, cyst area and total kidney area was performed using the FIJI image processing package of ImageJ (Fiji). Kidney cyst area was divided by whole kidney area to determine the cystic index^36,37^.

### Immunoblot analysis

Protein was extracted from renal tissue using radioimmunoprecipitation assay buffer (ThermoFisher) containing fresh protease and phosphatase inhibitor cocktail (1:100) (ThermoFisher). Equal amounts of protein (10–40 μg) were loaded onto 4–15% SDS–polyacrylamide gel electrophoresis gels, resolved by gel electrophoresis and transferred onto polyvinylidene difluoride membranes (Bio-Rad). Membranes were blocked in 5% bovine serum albumin or 5% milk in TBST and incubated overnight with primary antibody at 4 °C with gentle agitation. The primary antibodies used in these studies included PKM2 (4053; Cell Signaling), pPKMS-S37 (11456; SAB), β-catenin (8480; Cell Signaling) and β-actin (MA5-15739; ThermoFisher). Membranes were incubated with the appropriate horseradish peroxidase-conjugated secondary antibody before visualization using SuperSignal West Femto-enhanced chemiluminescence solution (34095; ThermoFisher) and the iBright FL 1000 (ThermoFisher). Optical density was determined using the ImageJ software from the NIH and Image Studio Lite from LI-COR. For PKM2 dimer immunoblot analysis, kidney lysates (2 mg/ml) were crosslinked using 0.025% glutaraldehyde for 3 min at 37 °C and Tris–HCl buffer added to a final concentration of 50 mM. After the addition of 2× Laemmli buffer, samples were separated by SDS–PAGE and transferred to a polyvinylidene difluoride membrane. After transfer, membranes were incubated in 0.4% paraformaldehyde in PBS for 30 min at room temperature, washed 3× in PBS and incubated overnight with total PKM2 antibody.

### Immunofluorescence

Kidneys were fixed overnight in 4% paraformaldehyde, washed with 1× PBS and then incubated in 30% sucrose for cryoprotection. The tissues were subsequently embedded and frozen in OCT medium (Tissue-Tek). Frozen sections (10 μm) obtained with a cryostat (Leica) were placed on slides, dried at room temperature, washed with 0.1% Triton x-100 in PBS, incubated with 5% BSA and then incubated overnight at 4 °C with antibodies against PKM2 (4053; Cell Signaling) or pPKMS-S37 (11456; SAB) and subsequently with anti-rabbit Alexa Fluor 647-conjugated secondary antibody (1:1000, Life Technologies). Sections were costained with Dolichos biflorus agglutinin (DBA; RL-1032; VectorLabs) or Lotus tetragonolobus lectin (LTL; FL-1321; VectorLabs) as well as 4,6-diamidino-2-phenylindole. Fluorescence was observed with a Zeiss LSM780 or LSM710 confocal microscope.

### Primary REC culture and spheroids

Two-week-old WT and *Glis3*-KO2 mice were killed and their kidneys immediately removed and placed in ice-cold PBS. The kidneys were thoroughly minced using an X-ACTO knife and placed into conical tubes of ice-cold digestion buffer consisting of 0.5 mg/ml type 1 collagenase and 0.25 mg/ml soybean trypsin inhibitor (ThermoFisher) dissolved in Hank’s Balanced Salt Solution. The tubes were placed in a 37 °C water bath and shaken for approximately 15 min. Digestion was followed by 2 min of gravity sedimentation; the top layer was subsequently transferred to a clean tube, digestion quenched by the addition of fetal bovine serum (FBS) and it was then centrifuged at 200 g for approximately 5 min. This process was repeated two times with the final resuspension using growth culture media consisting of advanced DMEM/F-12 Flex Media (ThermoFisher) supplemented with 1× MEM amino acids, 1× ITS-X, 0.5 mM sodium pyruvate, 2 mM glutamax, 10 mM glucose, 2% FBS and 1% penicillin–streptomycin (ThermoFisher) and plated onto 6- or 12-well plates. For small interfering (si)RNA-mediated knockdown of PKM2, three siRNA duplexes designed to target distinct regions of *Pkm2* were pooled together (SR416841; Origene). A scrambled siRNA was used as negative control (SR30004; Origene). A total of 2 days before spheroid culture, RECs were transfected with each duplex at 10 nM using SiTran 2.0 siRNA Transfection Reagent (TT320001; OriGene) according to the manufacturer’s protocol. For spheroid cultures, RECs were isolated from WT and *Glis3*-KO2 kidneys (*n* ≥ 4) and were grown on 100-mm dishes in growth medium until 80–90% confluent. Cells were trypsinized using TrypLE Express (Gibco #12605-010) and mixed with Matrigel (354277; Corning) to a final concentration of 160,000 cells/ml at a ratio of 70% Matrigel and 30% cells in media. The mixture was pipetted at ~50 µl onto prewarmed six-well plates for dome formation, resulting in approximately two domes per well. Plates were placed in a 37 °C incubator to polymerize for 15 min before warm complete media was added slowly to each well. Cells were allowed to grow for 24 h before administration of vehicle (0.1% DMSO) and compound 3K (1 μM) for 5–9 days in complete media without FBS. Spheroids were observed and imaged using the EVOS M7000 imager (Invitrogen). Images were obtained by taking *z*-stacks through each dome formation, and the size of spheroids of each group were analyzed using the FIJI image processing package of ImageJ.

### *Glis3* lentivirus

The *Glis3* lentiviral expression plasmid, pLVX-*Glis3*-mCherry, was derived from pLVX-mCherry-N1 (Clontech-Takara Bio) as described previously^38,39^. Primary kidney epithelial cells from WT and *Glis3*-KO2 mice were transiently transduced with pLVX-GLIS3-mCherry or pLVX-mCherry-N1 lentiviral vectors in 12-well plates. RNA was isolated 24 h after transduction for RT–qPCR analysis.

### Serum creatinine measurement

Blood was collected by cardiac puncture and transferred into BD Microtainer Blood Collection Tubes (365967; BD). Serum was separated according to the manufacturer’s instructions. Serum creatinine levels were measured using the Diazyme creatinine assay kit (DZ072B-KY1; Diazyme) following the manufacturer’s protocol. Creatinine concentrations are expressed as milligrams per deciliter (mg/dl).

### Statistical analysis and other software

All data are shown as mean ± s.e.m. When comparing two experimental groups, an unpaired, two-tailed *t*-test was used to determine statistical differences. A two-way analysis of variance followed by Tukey’s post hoc test was performed for comparisons of multiple groups. *P* < 0.05 was considered statistically significant. All statistical tests were performed using GraphPad Prism software (GraphPad Software). The BioRender.com online application was used for creating Figure 6i.

## Results

### GLIS3 regulates the transcription of several glycolytic and gluconeogenic genes

**Fig. 1:**
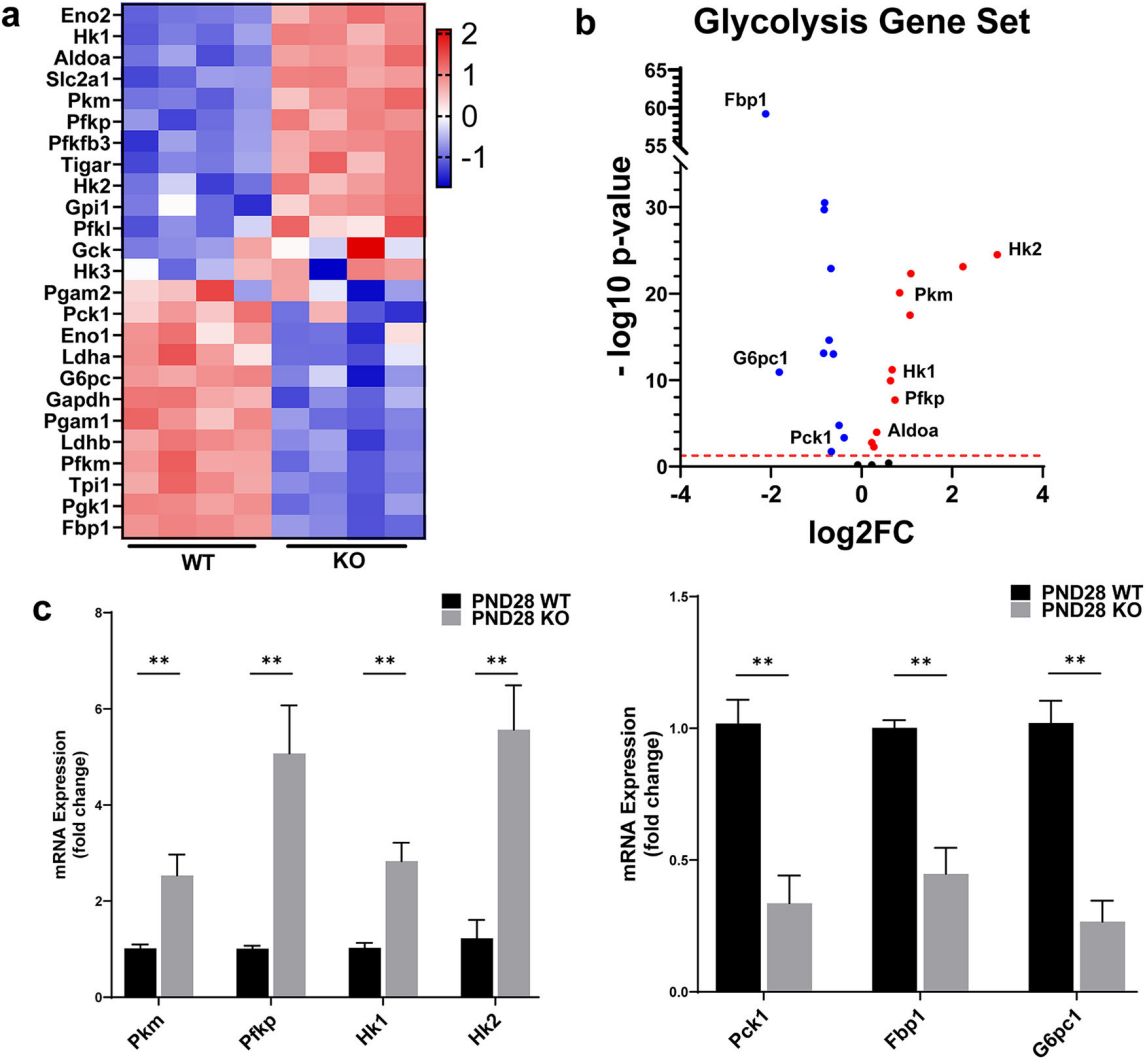
GLIS3 regulates the expression of several key glycolytic and gluconeogenic genes in the kidney. **a**, Heat map of glycolytic and gluconeogenic gene expression in kidneys from PND28 WT and *Glis3*-KO2 mice. Upregulated genes are represented in red and downregulated genes in blue. Expression values are shown as *z*-scores of the rlog-transformed values for each gene. **b**, Volcano plot of glycolytic and gluconeogenic genes differentially expressed in kidneys from PND28 *Glis3*-KO2 and WT mice. Several genes are indicated. **c**, RT–qPCR analysis of the expression of several glycolytic and gluconeogenic genes in kidneys from PND28 WT and *Glis3*-KO2 mice. Data are presented as mean ± s.e.m., *n* ≥ *5*. ^***^represents *P* < 0.001; ^**^represents *P* < 0.01; ^*^represents *P* < 0.05.

We previously reported that the increase in mitochondrial gene expression and dependence on oxidative phosphorylation for energy production during normal early postnatal kidney development are suppressed in GLIS3-deficient kidneys, which instead remain more reliant on aerobic glycolysis^11^. To obtain greater insights into the role of GLIS3 in the regulation of renal glycolysis, we analyzed the expression of several glycolytic and gluconeogenic genes in kidneys from WT and *Glis3*-KO2 mice. Transcriptomic analysis revealed that *Hk1/2*, *Pfkp*, *Aldoa* and *Pkm*, encoding enzymes with critical roles in the glycolytic pathway, were expressed at a higher level in PND28 *Glis3*-KO2 kidneys compared with PND28 WT kidneys, as shown in the heat map (Fig. 1a) and volcano plot (Fig. 1b). By contrast, several key gluconeogenesis-associated genes, such as *G6pc1*, *Fbp1* and *Pck1*, were expressed at a significantly lower level in PND28 *Glis3*-KO2 kidneys (Fig. 1a,b). The differential expression of several of these genes was supported by RT–qPCR analysis (Fig. 1c). The increased expression of *Hk1*, *Pfkp*, *Aldoa* and *Pkm* is consistent with our previous findings demonstrating a greater reliance on aerobic glycolysis and an increased conversion of pyruvate into lactate in *Glis3*-KO2 kidneys^11^. In addition, the reduced expression of *G6pc1*, *Fbp1* and *Pck1* probably impairs gluconeogenesis from pyruvate, thereby also contributing to the elevated lactate levels in *Glis3*-KO2 kidneys.

### GLIS3 directly regulates multiple key glycolytic and gluconeogenic genes

**Fig. 2:**
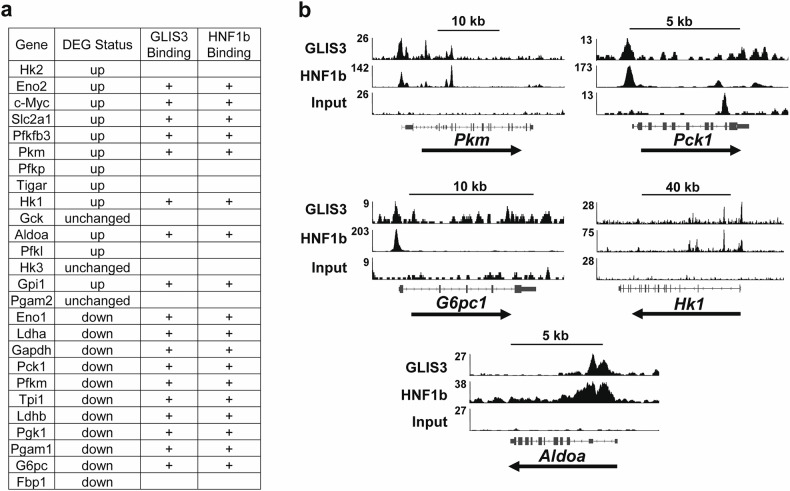
Transcriptional regulation of glycolytic and gluconeogenic genes by GLIS3. **a**, List of several differentially expressed glycolytic and gluconeogenic genes that contain GLIS3 and HNF-1B binding peaks (+) within ±5 kb of the corresponding transcription start site. **b**, Genome browser tracks of several glycolytic and gluconeogenic genes showing localization of GLIS3 and HNF-1B binding peaks within the same regulatory regions. Figure created in BioRender.

GLIS3 regulates gene transcription by binding to G/C-rich binding motifs in the regulatory regions of target genes^5,40^. Analysis of the GLIS3 cistrome identified GLIS3 binding sites (GLISBS) in several glycolysis and gluconeogenesis genes (Fig. 2a). The genome browser tracks in Fig. 2b show GLIS3 binding peaks associated with *Hk1*, *Pkm*, *Aldoa*, *G6pc1* and *Pck1*. These data indicate that GLIS3 regulates the transcription of these genes directly and further show that GLIS3 can function as a repressor and activator of gene transcription. We previously showed that GLIS3 regulates mitochondrial gene transcription in the kidney in coordination with other transcription factors, including HNF-1B^11^. Comparison of the GLIS3 and HNF-1B binding showed a large degree of overlap between the location of GLIS3 and HNF-1B binding peaks (Fig. 2a). Figure 2b shows the location of GLIS3 and HNF-1B binding peaks to the same regulatory region of several genes, consistent with the concept that GLIS3 regulates these genes in coordination with HNF-1B.

### Loss of GLIS3 function leads to the upregulation of *Pkm2* isoform in mouse kidney and in primary RECs

**Fig. 3:**
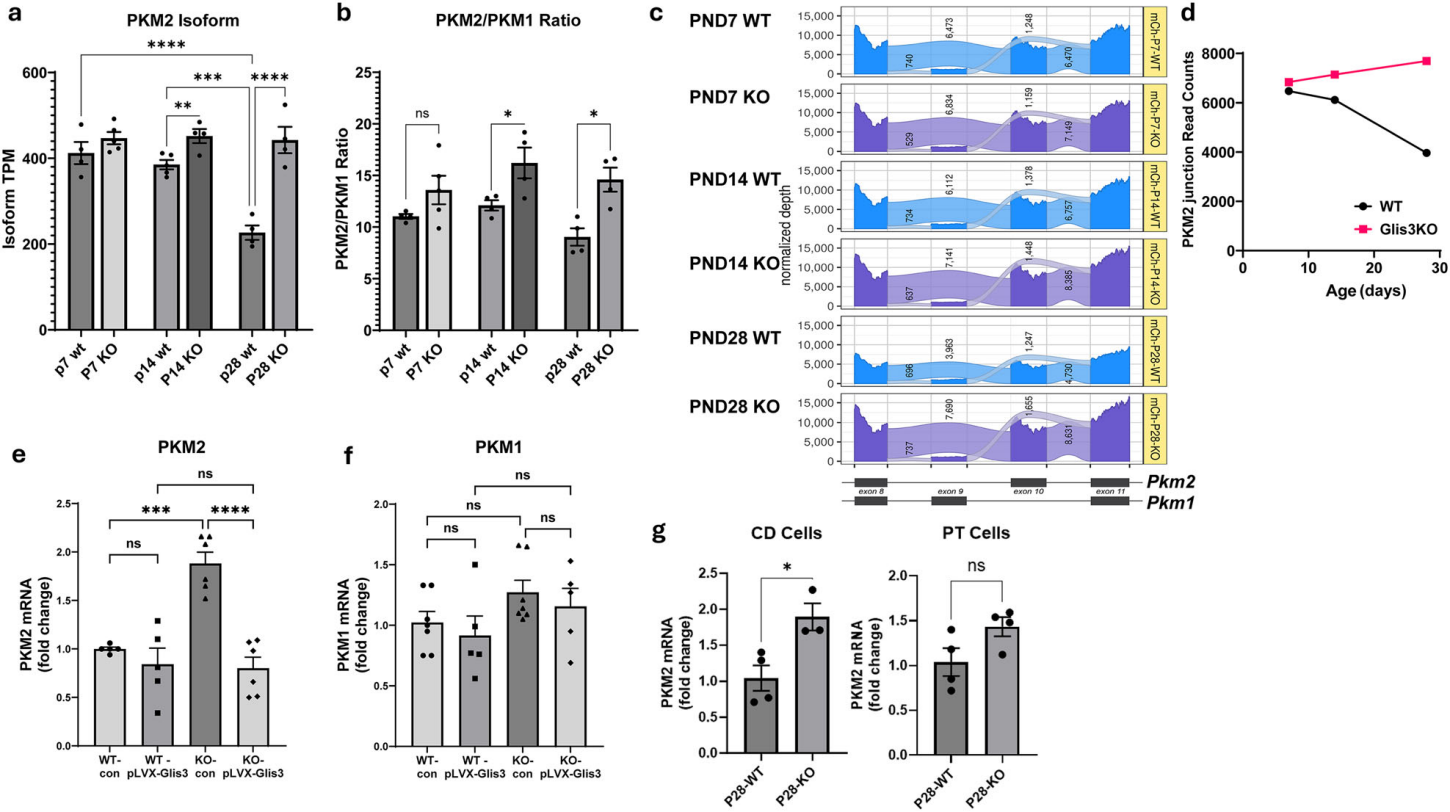
PKM2 transcripts are expressed at a higher level in *Glis3*-KO2 kidneys and primary *Glis3*-KO2 RECs compared with those of WT mice. **a**,**b**, Analysis of RNA-seq transcripts per million (TPM) values for PKM2 transcripts (**a**) and PKM2:PKM1 transcript ratio (**b**) in PND7, 14 and 28 WT and *Glis3*-KO2 kidneys. **c**, Sashimi analysis plot showing the alternatively spliced isoforms of PKM2 and PKM1. Exons (darker color) and splice junctions (lighter color) from WT are in blue and those from *Glis3*-KO2 are in purple. Splice junction read counts are numbered above their respective ribbons. STAR genomic alignment counts scaled to 300 M mappable reads per sample group labels the *y* axis, exon and splice junction coordinates are on the *x* axis and mRNA isoforms are shown on the bottom (exons in black and introns as lines). **d**, Analysis of the PKM2-specific splice junction read counts, plotted for visualization between PND7, 14 and 28 WT and *Glis3*-KO2 kidneys. **e**,**f**, Exogenous expression of GLIS3 in WT and *Glis3*-KO2 RECs decreased *Pkm2* mRNA expression (**e**) but did not change *Pkm1* mRNA expression (**f**). RECs were infected with Glis3 lentivirus for 36 h, and gene expression was analyzed by RT–qPCR. Data are presented as mean ± s.e.m., *n* ≥ *5*; ^****^P < 0.0001, ^***^P < 0.001; ^**^P < 0.01; ^*^P < 0.05. n.s., nonsignificant. **g**, RT–qPCR for *Pkm2* from isolated collecting duct (CD) and proximal tubule (PT) cells. Data are presented as mean ± s.e.m., *n* ≥ 3; ^*^P < 0.05.

We were particularly interested in the role that GLIS3 plays in the regulation of *Pkm* transcription and PKM protein activity, as PKM functions as a rate-limiting enzyme in the final step of glycolysis^41^ and has been reported to be important in modulating the metabolic fate of pyruvate between glycolysis and oxidative phosphorylation^27,42,43^. Through alternative splicing, *Pkm* produces two transcripts, *Pkm1* and *Pkm2*, containing exon 9 or 10, respectively, that encode PKM1 and PKM2, which have different functions^17,25,28,31,42,44^. Analysis of the expression of *Pkm* transcripts in WT and *Glis3*-KO2 kidneys showed that *Pkm2* transcripts and the *Pkm2*/*Pkm1* ratio are relatively higher in *Glis3*-KO2 kidneys at PND14 and PND28 compared with WT kidneys (Fig. 3a,b) indicating a selective upregulation of *Pkm2* expression. This was supported by analysis of the *Pkm2*-specific spliced junction read counts between exons 8 and 10 and exons 10 and 11, showing that in normal WT kidneys, these *Pkm2*-specific splice junctions decreased during PND7–PND28 (Fig. 3c–d). Conversely, the *Glis3*-KO2 kidneys exhibited higher *Pkm2*-specific read counts between exons 8 and 10 and exons 10 and 11 (Fig. 3c–d).

RT–qPCR analysis of *Pkm2* expression in primary cultures of RECs isolated from WT and *Glis3*-KO2 mouse kidneys showed that *Pkm2* mRNA was about twofold higher in the *Glis3*-KO2 RECs compared with the WT RECs (Fig. 3e). Regulation of *Pkm2* by GLIS3 was supported by the suppression of *Pkm2* expression in *Glis3*-KO2 RECs expressing exogenous GLIS3 (Fig. 3e). *Pkm1* expression was not significantly altered in *Glis3*-KO2 RECs compared with the WT RECs or by the expression of exogenous GLIS3 (Fig. 3f). These results suggest that GLIS3 acts as a negative regulator of *Pkm2* expression during early postnatal kidney maturation. Analysis of *Pkm2* RNA in isolated collecting duct and proximal tubule cells showed a nearly twofold higher expression in ductal cells from *Glis3*-KO2 kidneys compared with those of WT kidneys, while the small increase in tubule cells was not significant (Fig. 3g). We previously reported that *Glis3*-KO2 kidneys exhibit extensive cyst formation of the collecting ducts, while proximal tubules are only dilated^3^. Thus, increased *Pkm2* expression appears to correlate with prominent cystogenesis in collecting ducts.

### Loss of GLIS3 function causes an increase in PKM2 dimer formation and PKM2(pS37) and PKM2(pY105) levels

**Fig. 4:**
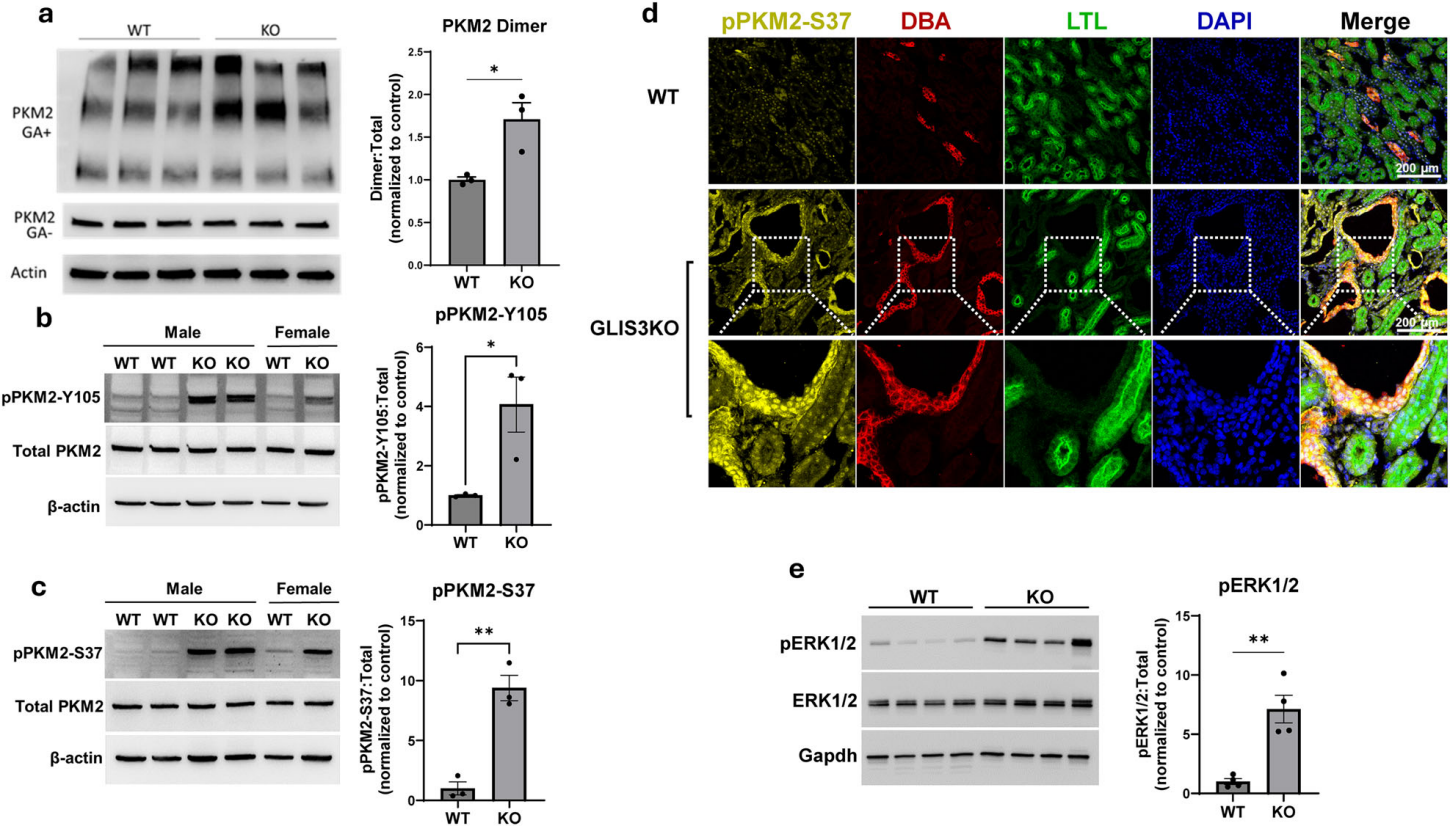
Levels of PKM2 dimers, PKM2(pS37) and PKM2(pY105) are increased in GLIS3-deficient kidneys. **a**, Immunoblot analysis of total PKM2 (GA−) and glutaraldehyde (GA+) crosslinked PKM2 expression. The relative level of PKM2 dimers were quantified by densitometric analysis. **b**, Immunoblot analysis of PKM2(pY105) and total PKM2 protein expression in WT and *Glis3*-KO2 kidneys. Protein expression was quantified by densitometric analysis. Data are presented as mean ± s.e.m., *n* = *3*; ^**^P < 0.01; *P < 0.05. **c**, Immunoblot analysis of PKM2(pS37) and total PKM2 protein expression in WT and *Glis3*-KO2 kidneys. Protein expression was quantified by densitometric analysis. Data are presented as mean ± s.e.m., *n* = *3*; ^**^P < 0.01; *P < 0.05. **d**, Representative images of PND28 WT and *Glis3*-KO2 kidney sections immunostained for PKM2(pS37) showing enhanced nuclear staining in renal cysts. The inlets denote the location of zoomed-in images. DBA marks collecting ducts and LTL marks proximal tubules. **e**, Immunoblot analysis of pERK1/2 and total ERK1/2 protein expression in WT and *Glis3*-KO2 kidneys. Protein expression was quantified by densitometric analysis. Data are presented as mean ± s.e.m., *n* = 4; ^**^P < 0.01.

Whether PKM2 is in a dimeric or tetrameric form is critical for its function in modulating aerobic glycolysis and oxidative phosphorylation. The tetrameric form has a high catalytic activity and favors oxidative phosphorylation, whereas the dimeric form typically exhibits a low catalytic activity and favors lactate production^28–32^. To investigate whether loss of GLIS3 function had any influence on the di/tetrameric levels of PKM2, we examined glutaraldehyde-crosslinked PKM2 from WT and *Glis3*-KO2 kidneys by western blot analysis. This analysis revealed that the relative level of the dimeric form of PKM2 and the dimer/total PKM2 ratio were increased in *Glis3*-KO2 kidneys (Fig. 4a). The formation of PKM2 dimers has been reported to be regulated by multiple posttranslational modifications^44^. PKM2 phosphorylation at tyrosine 105 (pY105) has been shown to promote dimer formation and aerobic glycolysis. Immunostaining and western blot analysis demonstrated that *Glis3*-KO2 kidneys contained higher PKM2(pY105) levels than WT kidneys (Fig. 4b), consistent with the observed increase in PKM2 dimers (Fig. 4a) and the higher reliance of GLIS3-deficient kidneys on aerobic glycolysis reported previously^11^. Phosphorylation of PKM2 at serine 37 (pS37) has also been shown to enhance the dimer state of PKM2 and allows its translocation to the nucleus where it participates in the regulation of gene transcription, including increased expression of *Pkm* itself^44–46^. Western blot analysis demonstrated a significantly higher level of PKM2(pS37) in *Glis3*-KO2 kidney compared with WT kidney (Fig. 4c). This was supported by immunofluorescence staining of PKM2(pS37) showing increased nuclear staining in *Glis3*-KO2 collecting duct cells within cysts compared with WT kidneys (Fig. 4d). Western blot analysis revealed that phosphorylated ERK1/2, a kinase responsible for PKM2(pS37) phosphorylation^44^, was also elevated in *Glis3*-KO2 kidneys (Fig. 4e). Together, increased phosphorylation of PKM2 at Y105 and S37 promote the formation of dimers, PKM2 nuclear localization and increased PKM2 expression and supports a role for PKM2 in metabolic reprogramming and cyst formation observed in the *Glis3*-KO2 kidneys.

### Effect of PKM2 knockdown and inhibition of PKM2 activity on the growth of renal cell spheroids

**Fig. 5:**
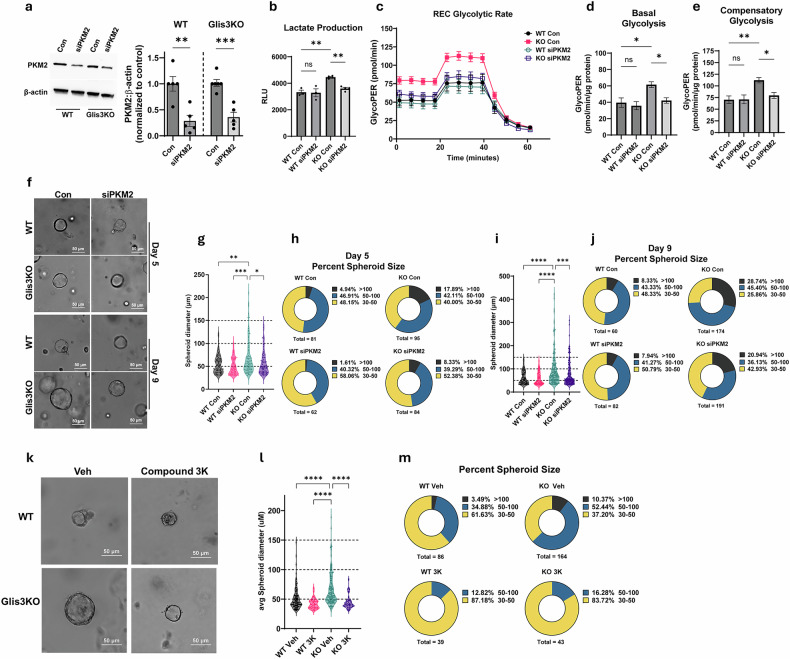
PKM2-KD and inhibition of its activity suppressed the growth of *Glis3*-KO2 REC spheroids. **a**, Immunoblot analysis of PKM2 protein levels in WT and *Glis3*-KO2 RECS 3 days after siRNA-mediated PKM2-KD. Protein expression was quantified by densitometric analysis. Data are presented as mean ± s.e.m., *n* = 5; ^***^P < 0.001; ^**^P < 0.01. **b**, Analysis of lactate production in media from primary WT and *Glis3*-KO2 RECs with or without PKM2-KD. **c**, Glycolytic rate was measured in primary WT and *Glis3*-KO2 REC mice with or without PKM2-KD using a Seahorse analyzer after sequential injections of rotenone/antimycin A and 2-DG. **d**,**e**, Basal (**d**) and compensatory (**e**) glycolysis were calculated and plotted (*n* = 3). **f**, Representative images of WT and *Glis3*-KO2 REC spheroids with and without PKM2-KD at 5 and 9 days. Bars indicate 50 μm. **g**, Violin plot showing the size (µm) distribution of the spheroids generated at day 5 from WT and *Glis3*-KO2 RECs with or without PKM2-KD (*n* ≥ 4). Each data point represents an individual spheroid measurement. ^*^P < 0.05, ^**^P < 0.01, ^***^P < 0.001. **h**, Spheroid images at day 5 were taken using the EVOS M7000, and spheroid diameter and number were analyzed using ImageJ and plotted according to size distribution—either 30–50, 50–100 or >100 μm. Total indicates the number of spheroids analyzed in each group (*n* = 39–164). **i**, Violin plot showing the size (µm) distribution of the spheroids generated at day 9 from WT and *Glis3*-KO2 REC mice with or without PKM2-KD (*n* ≥ 4). Each data point represents an individual spheroid measurement. *P < 0.05, ^**^P < 0.01, ^***^P < 0.001. **j**, Day 10 spheroid size distribution—either 30–50, 50–100 or >100 μm. Total indicates the number of spheroids analyzed in each group (*n* = 60–191). **k**, Representative image of the size of WT and *Glis3*-KO2 REC spheroids 5 days following treatment with vehicle (0.1% DMSO) or compound 3K (1 μM). Bars indicate 50 μm. **l**, Violin plot showing the size (µm) distribution of the spheroids generated from the RECs of WT and *Glis3*-KO2 kidneys (*n* ≥ 4). Each data point represents an individual spheroid measurement. ^****^P < 0.0001. **m**, Size distribution—30–50, 50–100 or >100 μm of WT and *Glis3*-KO2 REC spheroids with or without PKM2 inhibition. Total indicates the number of spheroids analyzed in each group *(n* = 39–164).

To obtain further evidence for a role of PKM2 in *Glis3*-KO2 renal cystogenesis, we examined the effect of both genetic knockdown of PKM2 and inhibition of PKM2 activity on cyst formation using a spheroid model of cultured primary RECs^47^. Primary *Glis3*-KO2 RECs formed larger spheroids than WT RECs in all experiments (Fig. 5f,k), consistent with the cystic phenotype observed in *Glis3*-KO2 kidneys. Notably, the *Glis3*-KO2 spheroids average diameter size increased from 69 µm at day 5 to 94.5 µm at day 9, whereas WT spheroids grew from 55 µm to 62 µm, recapitulating cyst progression and enlargement observed in mouse *Glis3*-KO2 kidneys (Fig. 5f–j).

First, we performed siRNA-mediated knockdown of PKM2 (PMK2-KD) in RECs derived from *Glis3*-KO2 and WT kidneys and confirmed a significant decrease in PKM2 protein levels in WT and *Glis3*-KO2 RECs (Fig. 5a). PKM2-KD markedly suppressed spheroid growth compared with control *Glis3*-KO2 cells at both day 5 and day 9 (Fig. 5f–j). To quantitate the effect PKM2-KD on spheroid size, we categorized the spheroids into three groups: 30–50 µm, 50–100 µm and >100 µm in diameter. At day 5, 17.89% of control *Glis3*-KO2 spheroids grew larger than 100 µm in diameter, compared with 8.33% in the PKM2-KD group (Fig. 5h). At day 9, 28.74% of the control *Glis3*-KO2 spheroids were larger than 100 µm in diameter compared with 20.94% in the PKM2-KD group, while 8.33% and 7.94%, respectively, of the control and PKM2-KD WT group were larger than 100 µm (Fig. 5j). In addition, PKM2-KD significantly decreased lactate production and attenuated both basal and compensatory glycolytic activity, as measured by Seahorse Bioanalyzer (Fig. 5b–e). As expected, *Glis3*-KO2 RECs exhibited higher baseline spheroid growth and glycolytic activity than WT RECs, consistent with their glycolytically reprogrammed phenotype, and PKM2-KD suppressed these increases in these parameters nearer to WT levels.

To complement these genetic studies, we next treated *Glis3*-KO2 spheroids with the PKM2 inhibitor, compound 3K^48^. This treatment greatly attenuated *Glis3*-KO2 REC spheroid size compared with vehicle-treated spheroids (Fig. 5k–m). A small reduction in spheroid size was also observed in compound 3K-treated WT REC spheroids (Fig. 5l). About 37%, 52% and 10% of the vehicle-treated *Glis3*-KO2 spheroids fell into the 30–50, 50–100 and >100 µm group, respectively, compared with 62%, 35% and 3% in vehicle-treated WT spheroids (Fig. 5m). After treatment with compound 3K, about 84% of *Glis3*-KO2 spheroids were 30–50 µm in diameter, with none exceeding 100 µm, while 87% of WT spheroids were 30–50 μm in diameter (Fig. 5m). The pharmacological inhibition of spheroid growth together with the suppression of both spheroid growth and glycolytic activity by PKM2-KD supports a regulatory role of PKM2 in metabolic reprogramming and progression of cystogenesis in GLIS3-deficient RECs.

### PKM2 inhibition reduces renal cyst progression in *Glis3*-KO2 mice

**Fig. 6:**
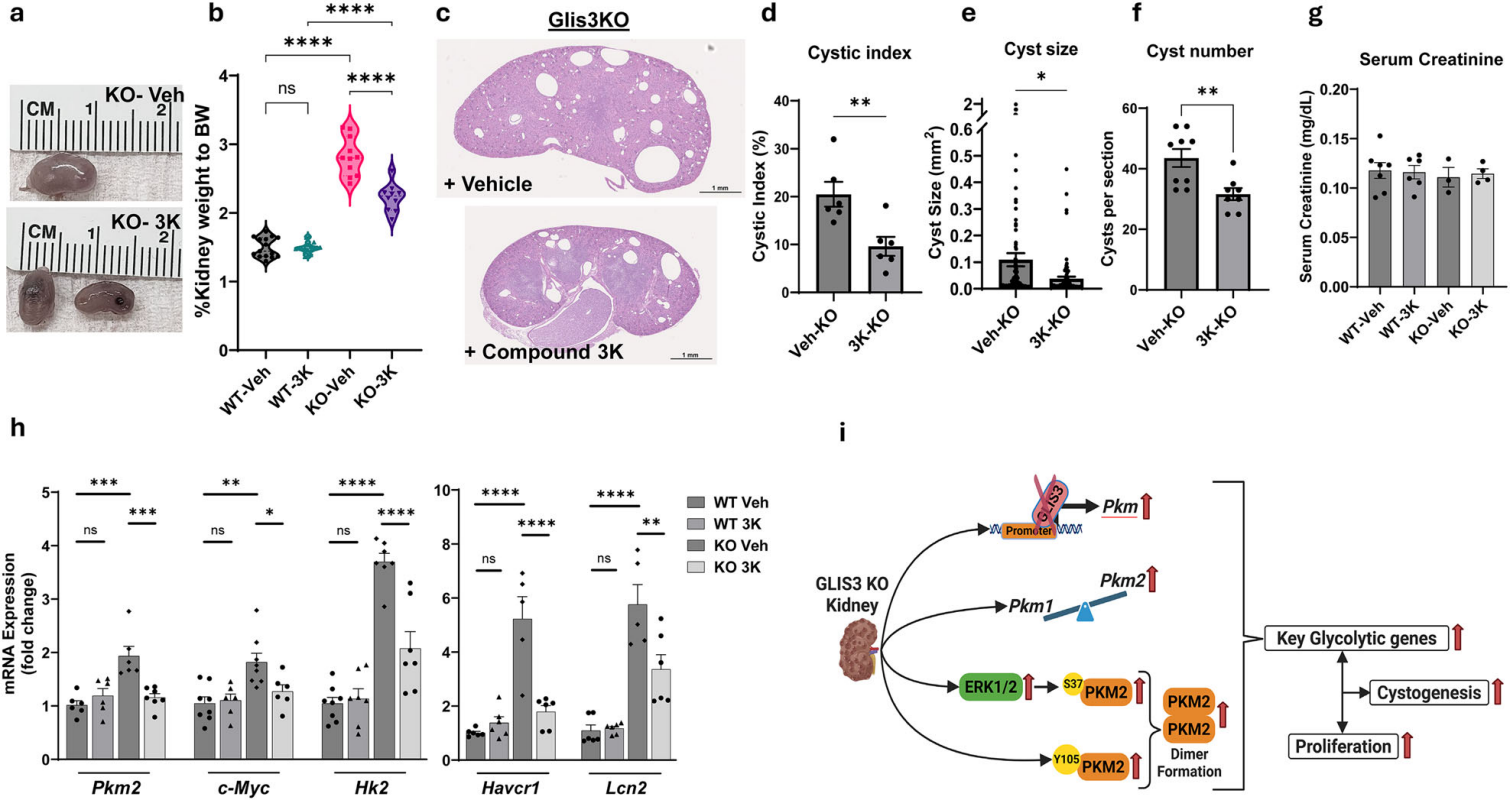
PKM2 inhibition suppresses renal cyst progression in *Glis3*-KO2 mice. **a**, Representative images comparing whole kidney size between Glis3-KO2 kidneys treated with vehicle or compound 3K are shown. **b**, Violin plot depicting the KW/BW ratio (%) for WT and *Glis3*-KO2 mice treated with vehicle or compound 3K. *n* ≥ 10; ^****^P < 0.0001. **c**, Representative hematoxylin and eosin-scanned images of kidney sections from *Glis3*-KO2 kidneys treated with vehicle or compound 3K. Bars indicate 1 mm. **d**, Comparison of cystic index between *Glis3*-KO2 kidneys treated with vehicle or compound 3K. Cystic index represents the percentage of renal tissue occupied by cysts. Data are presented as mean ± s.e.m., *n* = *6*. ^**^P < 0.01. **e**, Comparison of renal cyst size (mm^2^) between kidneys from *Glis3*-KO2 mice treated with vehicle or compound 3K. Data are presented as mean ± s.e.m., *n* = 6; ^*^P < 0.05. **f**, Comparison of the number of cysts per kidney section between *Glis3*-KO2 kidneys treated with vehicle or compound 3K. Data are presented as mean ± s.e.m., *n* = 8; ^**^P < 0.01. **g**, Comparison of serum creatinine levels (mg/dl) between WT and *Glis3*-KO2 mice treated with vehicle or compound 3K. **h**, RT–qPCR analysis of *Pkm2*, *c-Myc*, *Hk2*, *Havcr1* and *Lcn2* between WT and *Glis3*-KO2 mice treated with vehicle or compound 3K. Data are presented as mean ± s.e.m., *n* ≥ 5; ^****^P < 0.0001; ^***^P < 0.001; ^**^P < 0.01; ^*^P < 0.05. **i**, Schematic illustration depicting the relationship between loss of GLIS3 function, regulation of PKM2 and cystogenesis. GLIS3 deficiency enhances *Pkm* gene expression in kidneys with a preferential increase in the *Pkm2* isoform. Increased PKM2 phosphorylation at S37 and Y105 promotes dimer formation. PKM2-S37 phosphorylation is facilitated by increased pERK1/2 levels. Together, these events promote glycolysis, cell proliferation and cystogenesis in GLIS3-deficient kidneys. Figure 6i was created using BioRender.com.

To investigate the role of PKM2 in the progression of renal cystogenesis in vivo, we administered compound 3K (10 mg/kg) to *Glis3*-KO2 mice for 7 days starting at PND7. This time point was selected owing to the substantial increase in the number and size of renal cysts in *Glis3*-KO2 mice between PND7 and PND14. After 7 days of treatment, kidneys from compound 3K-treated *Glis3*-KO2 mice were significantly smaller in size (Fig. 6a) and the kidney weight/body weight ratio (KW/BW) was significantly reduced compared with the *Glis3*-KO2 vehicle-treated mice (Fig. 6b). As expected, following 7 days of vehicle control administration, the *Glis3*-KO2 mice exhibited a significantly higher KW/BW ratio compared with WT mice (Fig. 6b), while treatment with compound 3K reduced the average renal cyst size, cystic index (the percentage of cystic area) and number of cysts in *Glis3*-KO2 mice (Fig. 6d–f). At PND14, serum creatinine levels did not differ between WT and *Glis3*-KO2 mice, indicating that the cystogenesis was not yet severe enough to impact renal function (Fig. 6g). However, kidney injury markers can often precede kidney function decline; therefore, we measured the expression of the kidney injury markers *Havcr1* (KIM1) and *Lcn2* (NGAL). The expression of both genes was elevated about fivefold in vehicle-treated kidneys from *Glis3*-KO2 mice compared with those of WT controls (Fig. 6h). Treatment with compound 3K significantly reduced the expression levels of *Havcr1* and *Lcn2*, suggesting that PKM2 inhibition attenuates cystogenesis-associated injury (Fig. 6h). In addition, PKM2 inhibition suppressed the increase in *Pkm2*, *c-Myc* and *Hk2* expression in *Glis3*-KO2 kidneys, consistent with a reduced reliance on aerobic glycolysis and partial restoration of a more normal metabolic state (Fig. 6h). Collectively, these results demonstrate that PKM2 inhibition can significantly attenuate renal cyst progression and support a role for PKM2 in renal cystogenesis in *Glis3*-KO2 mice (Fig. 6i).

## Discussion

Loss of GLIS3 function in humans and mice causes a multiorgan phenotype that includes ARPKD^2,3,7,11,12,49^. During the first postnatal month, mouse kidneys undergo further maturation, as indicated by increased expression of functional genes, including genes encoding various transporters. In addition, this period is associated with a metabolic shift, during which oxidative phosphorylation becomes the major source of energy generation rather than aerobic glycolysis^15,16,20^. In PKD, including that in GLIS3-deficient mice, energy metabolism is dysregulated and RECs remain more reliant on aerobic glycolysis (referred to as metabolic reprogramming)^11,15,16,20^. We recently reported that GLIS3 regulates bioenergetic processes during early postnatal kidney development in part via the transcriptional regulation of several genes involved in oxidative phosphorylation^11^. Whether GLIS3 also has a role in regulating gene transcription of glycolytic and gluconeogenic pathways was not determined.

To study whether GLIS3 has any role in controlling glycolysis by regulating the expression of genes in the glycolytic pathway, we performed transcriptome and cistrome analysis. Transcriptome analysis showed that several genes critical in glycolysis (*Hk1*, *Pfkp*, *Aldoa* and *Pkm*) were expressed at a higher level in PND28 *Glis3*-KO2 kidneys than in WT kidneys, whereas several important gluconeogenic genes (*Pck1*, *G6pc* and *Fbp1*) were expressed at a reduced level (Fig. 1a). ChIP-seq analysis identified GLIS3 binding peaks in several differentially expressed, glycolysis- and gluconeogenesis-related genes (Fig. 2). These findings suggest that GLIS3 directly regulates the transcription of these genes by binding GLISBS in their regulatory regions. This analysis further showed that GLIS3 binding peaks were both associated with genes that were upregulated or downregulated in *Glis3-*KO2 kidneys, indicating that GLIS3 functions both as repressor and activator of gene transcription. This conclusion is consistent with our previous studies in thyroid follicular cells and pancreatic beta cells^6,40^. In addition to the GLISBS consensus binding site, Homer analysis identified a consensus HNF-1B binding site, suggesting that GLIS3 and HNF-1B binding sites are frequently localized within the same regulatory region of target genes^11^. This was supported by genome browser tracks of *Pkm*, *Hk1*, *Pck1*, *G6pc1* and *Aldoa*, which revealed a large degree of overlap between the location of GLIS3 and HNF-1B binding peaks (Fig. 2b), indicating that these genes are probably coordinately regulated by GLIS3 and HNF-1B. The latter is of particular interest because of the similarities in the cystic phenotype between GLIS3- and HNF-1B-deficient kidneys. Future studies using single-cell transcriptomics with *Hnf1b*-KO kidneys need to confirm whether these HNF-1B target genes are indeed differentially expressed and regulated by HNF-1B^3,50^.

The regulation of *Pkm* by GLIS3 was particularly important because it encodes PKM, a rate-limiting enzyme in the final step of glycolysis, that also plays a critical role in modulating aerobic glycolysis and oxidative phosphorylation. Through alternative splicing, *Pkm* produces two transcripts, *Pkm1* and *Pkm2*, which encode two functionally different proteins, PKM1 and PKM2^17,25,28,31,42,44^. Analysis of the *Pkm* transcripts showed that *Pkm2* transcripts and the *Pkm2*/*Pkm1* ratio were higher in *Glis3*-KO2 kidneys, thus favoring *Pkm2* expression (Fig. 3a–c). The activity of PKM2 is dependent on whether it is in a dimeric or tetrameric complex with the tetrameric complex favoring oxidative phosphorylation and the dimeric state aerobic glycolysis. We show that *Glis3*-KO2 kidneys contained higher levels of PKM2 dimers than WT kidneys (Fig. 4a), which is consistent with previous data showing that *Glis3*-KO2 renal cells are more reliant on aerobic glycolysis^11^. Whether PKM2 is in a dimeric or tetrameric state is under complex control that includes posttranslational modifications and interaction with metabolites, such as fructose 1,6-biphosphate (FBP)^29,51^. Interaction with FBP stabilizes the formation of PKM2 tetramers, while PKM2 phosphorylation at Y105 releases FBP, leading to an increase in PKM2 dimers. We showed that *Glis3*-KO2 kidneys express higher levels of PKM2(pY105), consistent with the observed increase in PKM2 dimers (Fig. 4b). We also demonstrated that *Glis3*-KO2 kidneys express higher levels of PKM2(pS37) (Fig. 4c). Phosphorylated ERK1/2, which has been reported to catalyze PKM2(pS37) phosphorylation^44^, was also found to be elevated in *Glis3*-KO2 kidneys (Fig. 4e) suggesting that it mediates the increase in PKM2(pS37) in *Glis3*-KO2 kidneys. Several studies have demonstrated that phosphorylation at S37 induces PKM2 nuclear translocation where it functions as a transcriptional co-activator of various genes^29,51^, including *Pkm* and *c-Myc*. Although a direct PKM2–*c-Myc* regulatory link in *Glis3*-KO2 kidneys was not examined, the increased expression of these genes (Fig. 6h), together with the elevated levels of PKM2(pY105) and PKM2(pS37), PKM2 dimer formation and PKM2 nuclear localization (Fig. 4), is consistent with the concept that PKM2 may act as a co-activator. These combined changes probably contribute to the upregulation of these genes and to the greater reliance on aerobic glycolysis (metabolic reprogramming) in *Glis3*-KO2 kidneys. In addition, the colocalization of GLIS3 and HNF-1B binding peaks within *Pkm* and *c-Myc* (Fig. 2b and Supplementary Fig. 1) suggest that regulation of these genes is multifactorial in GLIS3-deficient renal cells. Future studies using transgenic mice carrying phosphorylation-related mutations at Y105 and S37 would help clarify their specific role in kidney maturation and cystogenesis.

As has been reported for various cancer cells^24,32,52,53^, this metabolic shift probably promotes proliferation of *Glis3*-KO2 RECs, thereby contributing to cystogenesis. The latter is supported by data showing that primary *Glis3*-KO2 RECs formed larger spheroids in vitro than WT RECs (Fig. 5f–l) and is consistent with the concept that elevated PKM2 is part of metabolic reprogramming and contributes to increased glycolysis and cell proliferation. Indeed, PKM2-KD in *Glis3*-KO2 RECs suppressed spheroid growth, reduced lactate production and decreased both basal and compensatory glycolysis (Fig. 5b–i), further strengthening a regulatory role of PKM2 in the changes in glycolysis and proliferation associated with GLIS3-deficient RECs.

The role of PKM2 dimers in promoting metabolic reprogramming, cell proliferation and cystogenesis raised the question whether inhibition of PKM2 would suppress the growth and expansion of *Glis3*-KO2-induced cysts. In agreement with the PKM2-KD studies, pharmacological inhibition of PKM2 with compound 3K resulted in a similar suppression of spheroid growth. This was supported by data showing that treatment of spheroids of *Glis3*-KO2 RECs with compound 3K significantly attenuated the increased growth and size of *Glis3*-KO2 REC spheroids, with a limited effect on the growth of WT spheroids (Fig. 5j–l). This growth inhibition is consistent with the observed reduced proliferation in cancer cells treated with compound 3K^48^. We further demonstrated that administration of compound 3K to PND7 kidney-selective Glis3-knockout (*Glis3*-Pax8Cre) pups for 7 days reduced kidney size, cystic index and the number of cysts compared with vehicle-treated mice (Fig. 6), indicating that inhibition of PKM2 has a protective effect against cystogenesis.

In summary, our study provides new insights into the pathogenesis of renal cyst formation in GLIS3-deficient kidneys. We identified GLIS3 as a direct transcriptional regulator of several glycolytic genes and show that GLIS3 regulates these genes in coordination with HNF-1B, another key regulator of kidney functions^50^. We demonstrate that the elevated expression of glycolytic genes and increased formation of PKM2 dimers, mediated by increased phosphorylation at Y105 and S37, in GLIS3-deficient kidneys leads to a greater reliance on aerobic glycolysis (metabolic reprogramming) that promotes cell proliferation and cystogenesis (Fig. 6i). Importantly, both genetic knockdown and pharmacological inhibition of PKM2 significantly attenuated the cystic phenotype and expansion in *Glis3*-KO2 RECs. These findings support a role for PKM2 in the regulation of the dynamics between aerobic glycolysis and oxidative phosphorylation. Importantly, the inhibition of cyst growth and cystogenesis in GLIS3-deficient kidneys by a PKM2 inhibitor suggests that targeting PKM2 may provide a promising therapeutic strategy to slow or prevent the progression of cystic kidney disease.

## Supplementary information

Supplementary Information

## Data Availability

The RNA-seq and ChIP-seq datasets of *Glis3*-KO2 mouse kidneys that were used for supporting the findings of this study were deposited in Gene Expression Omnibus under the GEO accession nos. GSE240074 and GSE240072.
