# Supplementary material for: Regulation of PKM2 expression and function by GLIS3 during metabolic reprogramming in polycystic kidneys
Source: Exp Mol Med. 2026 Mar 13;58(3):932–41. doi: 10.1038/s12276-026-01676-5 (PMC13048982; doi:10.1038/s12276-026-01676-5)
Supplement: Supplementary file 1 — Supplementary Information [file 12276_2026_1676_MOESM1_ESM.pdf]

Regulation of PKM2 expression and function by GLIS3 during metabolic reprogramming in polycystic kidneys

Justin B. Collier[1], Hong Soon Kang[1], Sara A. Grimm[2], Tanushree Mukherjee[1],

Chitrangda Srivastava[1], and Anton M. Jetten[1]*

[1]Cell Biology Group, Immunity, Inflammation and Disease Laboratory
[2]Integrative Bioinformatics
National Institute of Environmental Health Sciences
National Institutes of Health
Research Triangle Park, NC 27709, USA

* To whom correspondence should be addressed
E-mail: jetten@niehs.nih.gov

Supplementary Information

Contents:

Supplementary Figures

Supplementary Figure 1. Genome browser track of c-Myc showing localization of GLIS3 and HNF1B binding peaks within the same regulatory region.

Supplementary Tables

Supplemental Table 1. List of mouse primers used for qRT-PCR

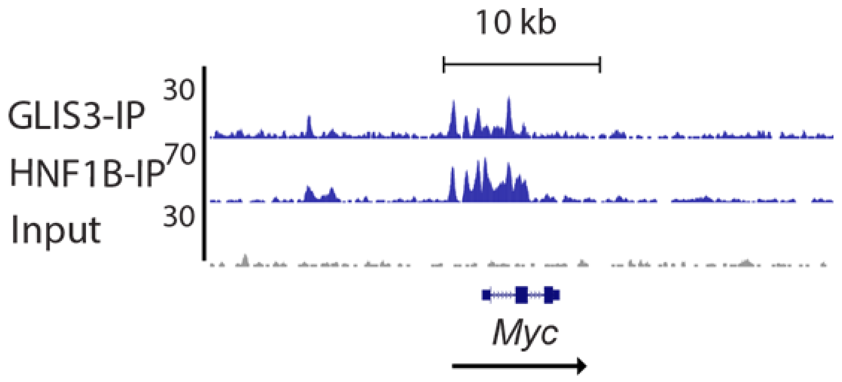

Supplementary Fig. 1. Genome browser track of *c-Myc* showing localization of GLIS3 and HNF1B binding peaks within the same regulatory region.

Name	F	R
Pkm	TGACACCTTCCTGGAACACA	TTCAGCATCTCCACAGATCG
Pkm1	GTCTGGAGAAACAGCCAAGG	TCTTCAAACAGCAGACGGTG
Pkm2	GTCTGGAGAAACAGCCAAGG	CGGAGTTCCTCGAATAGCTG
Pfkp	ACTCCGAGGAAGGCGTTTTG	GGAGACAGTAGCAGGAACCAT
Pck1	CGATGACATTGCCTGGATGAAG	TCTTCACTGAGGTGCCAGGAG
Fbp1	ATGCCATCATAATCGAACCTGAG	GGACACAAGGCAGTCAATGTTG
G6pc1	ATGAACATTCTCCATGACTTTGGG	GACAGGGAACTGCTTTATTATAGG
c-Myc	GCGACTCTGAAGAAGAGCAAGA	GCACCTCTTGAGGACCAGTG
Hk1	GGGACTATGACGCTAACATT	CCAGTGCCAATGATCAGG
Hk2	TGATCGCCTGCTTATTCACGG	AACCGCCTAGAAATCTCCAGA
Havcr1	GCATCTCTAAGCGTGGTTGC	TCAGCTCGGGAATGCACAA
Lcn2	GCTACAATGTCACCTCCATCCTG	CTGGAGCTTGGAACAAATGTTCTG
Actb	AGCAGTTGGTTGGAGCAAACATCC	GTGAGGGACTTCCTGTAACCACTT

Supplementary Table 1. List of mouse primers used for qRT-PCR
